# Cognitive outcomes after endovascular thrombectomy in ischemic stroke: a systematic review

**DOI:** 10.3389/fmed.2026.1787129

**Published:** 2026-05-11

**Authors:** Wiktoria Balcerzak, Gabriela Początek, Anetta Lasek-Bal, Agnieszka Gorzkowska

**Affiliations:** 1 Department of Neurology, Upper Silesian Medical Center of the Medical University of Silesia, Katowice, Poland; 2 Doctoral School of Medical and Health Sciences, Medical University of Silesia, Katowice, Poland; 3 Department of Neurology, School of Health Sciences, Medical University of Silesia, Katowice, Poland

**Keywords:** acute ischemic stroke, bridging therapy, cognitive impairment, endovascular therapy, neuropsychology, reperfusion, thrombectomy

## Abstract

**Background/objective:**

Endovascular thrombectomy (EVT) is the standard treatment for acute ischemic stroke due to large-vessel occlusion (LVO), but its impact on cognition is less clearly defined despite the high prevalence of post-stroke cognitive impairment. We systematically reviewed randomized controlled trials and observational studies reporting objectively measured cognitive outcomes after EVT in adults with acute ischemic stroke.

**Methods:**

Following PRISMA 2020, a systematic search of PubMed, Embase, the Cochrane Library, and Scopus was conducted between 1 March and 31 October 2025 for studies published from 1 January 2015 to 31 October 2025. Eligible studies reported objectively measured post-stroke cognitive outcomes after EVT, with or without intravenous thrombolysis (IVT). Due to substantial heterogeneity in cognitive assessment tools, outcome definitions, and follow-up time points, quantitative pooling was not feasible and results were therefore synthesized narratively.

**Results:**

Twenty-three studies (∼3,300 participants; 3 RCTs, 20 observational) met the inclusion criteria. Across most comparative studies, EVT was associated with better cognitive outcomes than IVT or best medical therapy, typically yielding on average 1–4 point higher scores on the Montreal Cognitive Assessment (MoCA) or Mini-Mental State Examination (MMSE) at early to mid-term follow-up. The most consistent benefits were observed in executive functions, with more variable but favorable effects on memory. Worse cognitive outcomes were linked to larger infarct volume, combined gray–white matter involvement, territorial infarct patterns, and longer time to reperfusion. Exploratory proteomic work suggested associations between inflammatory and neurotrophic markers and cognitive recovery. Despite these benefits, post-stroke cognitive impairment remained frequent.

**Conclusion:**

Endovascular thrombectomy, particularly when performed rapidly with successful reperfusion, is associated with more favorable cognitive outcomes, especially in global cognition and executive function. However, persistent cognitive deficits remain common, highlighting the need for standardized cognitive assessment and longer-term follow-up in future EVT studies.

**Systematic Review Registration:**

https://www.crd.york.ac.uk/PROSPERO/view/CRD420251156363, identifier CRD420251156363.

## 1 Introduction

Acute ischemic stroke (AIS) remains a leading cause of death and long-term disability worldwide (1). Rapid reperfusion is essential for improving outcomes–every hour of delay in treatment significantly reduces the likelihood of functional independence (2). Intravenous thrombolysis (IVT) is recommended for eligible patients within 4.5 h of symptom onset (3), while endovascular thrombectomy (EVT) has transformed the management of large-vessel occlusion (LVO) stroke. Pivotal trials have established EVT as superior to medical therapy alone and have extended the therapeutic window for selected patients based on advanced imaging (4). When appropriate, IVT and EVT are combined as bridging therapy, although recent evidence has yielded mixed results regarding its superiority over direct EVT (2).

Cognitive impairment is one of the most common and disabling consequences of stroke, affecting more than two-thirds of survivors and contributing substantially to long-term dependency (5–7). These deficits range from mild impairment to post-stroke dementia (8) and may affect attention, memory, executive functioning, language, and visuospatial abilities (9, 10). Their severity is shaped by lesion characteristics, treatment timeliness, and individual premorbid factors.

Despite the clear functional benefits of EVT, its specific impact on cognition remains less well established. Available studies have reported heterogeneous findings: several comparative analyses found better global cognition and/or executive performance after EVT than after intravenous thrombolysis or best medical therapy (11–14), whereas others reported more limited between-group differences or neutral findings, particularly when comparing EVT strategies or selected patient subgroups (15, 16). These inconsistencies may reflect substantial heterogeneity in study design, patient selection, stroke location and severity, infarct morphology, reperfusion success, treatment timing, cognitive instruments, and follow-up duration (11–13, 15, 17). In addition, cognition has rarely been prespecified as a primary endpoint in EVT studies (13, 14). Therefore, the extent to which EVT improves cognition beyond functional recovery remains uncertain.

This systematic review therefore aims to synthesize current evidence on cognitive outcomes after EVT, compare EVT with IVT or best medical therapy, identify cognitive domains most affected, and examine clinical, imaging, and biological predictors of cognitive recovery. A consolidated understanding is essential, given the central role of cognitive function in long-term independence.

## 2 Materials and methods

### 2.1 Literature review and eligibility

A systematic literature review was conducted in accordance with the PRISMA 2020 statement (Preferred Reporting Items for Systematic Reviews and Meta-Analyses) (18) between 1 March 2025 and 31 October 2025. A comprehensive search of PubMed, Embase, the Cochrane Library, and Scopus was performed for articles published in English between 1 January 2015 and 31 October 2025.

The search strategy combined three main concepts using Boolean operators: (1) ischemic stroke; (2) mechanical thrombectomy; and (3) cognitive outcomes or neuropsychological assessment. Search terms included both controlled vocabulary (MeSH and Emtree) and free-text keywords related to ischemic stroke, thrombectomy, cognition, and specific neuropsychological tests (e.g., MMSE, MoCA, TMT, Stroop, CVLT, FAB).

The search was limited to human studies published in English. Gray literature, conference abstracts, dissertations, and non-peer-reviewed sources were excluded. The full search strings for each database are provided in Supplementary Table 1.

The review protocol was registered in PROSPERO (CRD420251156363).

Eligibility criteria were defined using a PICOS framework:

- Population (P): Adults (≥18 years) with AIS.
- Intervention (I): EVT, with or without IVT.
- Comparator (C): IVT alone, best medical therapy, standard medical care, or no reperfusion therapy; single-arm EVT cohorts were also included when reporting prognostic factors for cognition.
- Outcomes (O): Objectively measured post-stroke cognitive function, assessed using validated cognitive instruments (e.g., MoCA, MMSE, TMT, RBANS).
- Study design (S): Randomized controlled trials (RCTs) and observational studies published in peer-reviewed journals from 2015 onward.

We excluded studies that lacked objective cognitive testing, did not specify acute-phase stroke treatment, focused on non-thrombectomy interventions or non-stroke populations, involved animals or preclinical models, were study protocols, or were not original research (e.g., reviews, editorials, surveys, case reports).

### 2.2 Data extraction, synthesis, and quality assessment

Three reviewers independently screened all titles, abstracts, and full texts, resolving disagreements through discussion. Extracted data included study design, sample characteristics, stroke and treatment details, co-interventions, cognitive assessment tools and timing, primary cognitive outcomes, and imaging or biomarker correlates. Owing to substantial heterogeneity in cognitive measures, follow-up intervals, comparators, and effect-size reporting, a meta-analysis was not feasible; findings were therefore narratively synthesized and summarized in tables. Risk of bias was assessed independently using RoB 2 for randomized trials and the Newcastle–Ottawa Scale for observational studies, with discrepancies resolved by consensus or consultation with a fourth reviewer. Certainty of evidence across cognitive domains was graded using the GRADE framework, considering study limitations, inconsistency, indirectness, imprecision, and potential publication bias.

## 3 Results

### 3.1 Study selection

**FIGURE 1:**
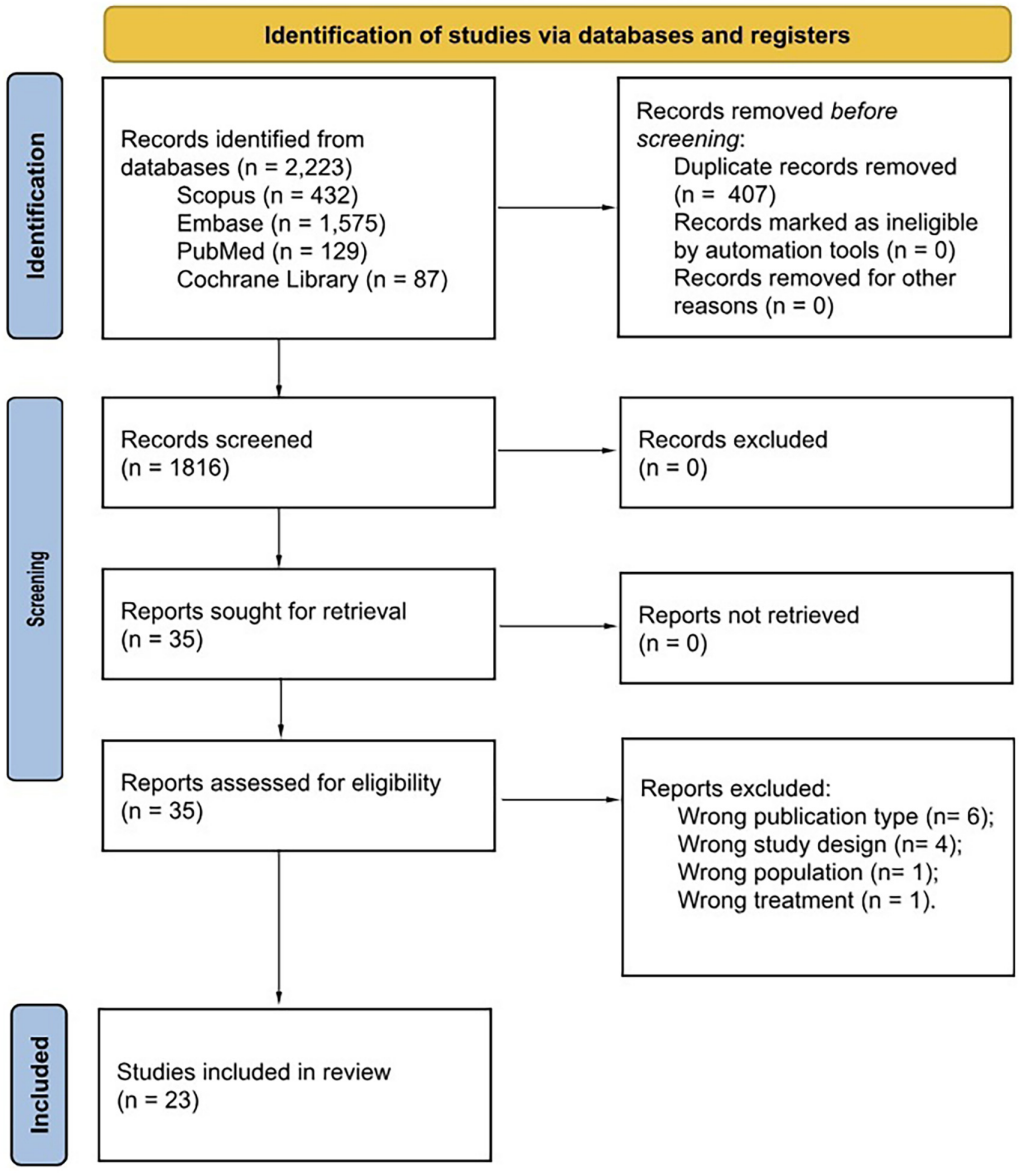
Preferred Reporting Items for Systematic Reviews and Meta-Analyses (PRISMA) flow diagram outlining the study identification, screening, eligibility assessment, and inclusion process.

A total of 2223 records were identified through database searches. After removing 407 duplicates, 1816 records remained for title and abstract screening. Of these, 1781 were excluded based on titles and abstracts. A total of 35 full-text reports were sought for retrieval and assessed for eligibility. After applying inclusion and exclusion criteria, 23 studies were included in the qualitative synthesis. The study selection process is illustrated in the PRISMA 2020 flow diagram (Figure 1).

### 3.2 Study characteristics

This review included 23 studies–RCTs and observational cohorts–evaluating cognitive outcomes after acute stroke treatments (11–17, 19–35). Sample sizes ranged from small prospective cohorts (≈25–60 patients) (19–21) to one large multicenter study (*n* = 1026) (17). Most focused on anterior circulation LVO (ICA, M1/M2) (11, 12, 16, 22–24), though some included PCA strokes (25), vertebrobasilar occlusions (12), or mixed territories (15, 26).

Interventions primarily involved EVT or bridging therapy (EVT + IVT) (11, 23, 28), with several comparisons against IVT alone or best medical therapy (12–15, 22). Other studies examined adjunctive pharmacologic strategies (tirofiban, butylphthalide) (31, 32), dual antiplatelet therapy duration (30), treatment timing (27, 28), and proteomic/inflammatory markers related to recovery (e.g., IGFBP3, MCP-1, COMP) (19, 29, 33). Some also assessed infarct morphology–gray-matter vs. mixed lesions, territorial vs. scattered patterns–as predictors of cognitive outcomes (14, 17).

Cognition was most commonly measured with MoCA or MMSE (12, 13, 15, 19, 21, 24, 28–31, 33), supplemented by domain-specific tests such as TMT-A/B, Stroop, RAVLT, ROCF, RBANS, and language measures (AQ, WAB, BNT) (11, 12, 20, 21, 25, 26). Follow-up varied, though 3-months assessments predominated, with only a few studies reporting outcomes beyond 12 months (14, 27).

Overall, these studies offer a broad overview of cognitive trajectories after EVT, IVT, and adjunctive therapies, highlighting the roles of treatment timing, infarct morphology, and inflammatory/neurotrophic signaling in shaping post-stroke cognitive recovery. Tables 1, 2 summarize key study characteristics and findings.

**TABLE 1:** Study characteristics.

References	Sample size (*n*)	Groups/intervention type	Stroke location	Cognitive assessment tools
Lattanzi et al. (11)	88	EVT + IVT vs. IVT	Anterior circulation LVO (ICA/M1/M2/A1/A2)	Stroop, TMT-A/B, Digit Span F/B, CPM, ROCF (Copy/Imm/Del), RAVLT (Imm/Del)
Humphrey et al. (12)	62	EVT vs. BMT (tPA + conservative combined)	EVT: ICA/M1/M2/BA; BMT: smaller vessels	TOPF, MoCA, WAIS-IV, WMS-IV, RAVLT, BVMT, BIT, BNT, COWAT, TMT, D-KEFS CWIT, TOL, HADS, FSS, BI, NEADL, FrSBe
Maglinger et al. (19)	52 (28 at 90 days)	EVT	ELVO (ICA, MCA, BA)	MoCA, proteomic analysis (Olink panel)
Ospel et al. (17)	1026	EVT	Anterior circulation LVO (infarct phenotype analysis)	MoCA (90 d), SNAP, BNT
Xu et al. (22)	90	EVT guided by multimodal CT/MRI vs. IVT	Mild–moderate anterior circulation stroke (NIHSS ≤ 15; ASPECTS ≥ 5)	MoCA, MMSE, HIS
Hazelwood et al. (29)	61 + 13 matched pairs	EVT vs. CVD controls	ELVO	MoCA (90 d), proteomic analysis
D’Netto et al. (20)	25	EVT (with or without IVT)	Anterior circulation unilateral stroke	RBANS, TMT-A/B, Brixton, WAB, FOIS
Costa Novo et al. (27)	44	EVT (with or without IVT; TICI ≥ 2b)	Right anterior circulation stroke	ACE-R, ARWMC
Li et al. (30)	147	EVT (1-month vs. 3-months DAPT)	LVO (MCA/ICA)	MoCA
Bao et al. (31)	73	EVT + adjunctive therapy vs. EVT	ICA-T/M1/M2/A2	MMSE, MoCA
Ye (34)	60	EVT (TREVO) vs. IVT	Mixed vascular territories	MoCA
Chen et al. (32)	102	EVT + tirofiban vs. EVT	ICA/MCA	MMSE
McLouth et al. (33)	81	EVT; proteomic analysis	ELVO	MoCA
Pu et al. (23)	114	EVT + IVT vs. IVT	LVO (“critical” vs. “non-critical” infarct location)	MMSE
Chen et al. (24)	169	EVT + BMT vs. BMT	ICA/MCA	MMSE, MoCA
Regush et al. (26)	96	EVT	Mixed vascular territories	TMT-A, Stroop, Luria
Joundi et al. (13)	315	EVT vs. BMT	ICA/M1; ASPECTS 6–10	MoCA, SNAP, BNT, TMT-A/B
Humphrey et al. (15)	82	EVT vs. IVT vs. BMT	Mixed vascular territories	MoCA
Guglielmi et al. (21)	43	EVT (TICI ≥ 2b)	Basal ganglia infarcts	MoCA, RAVLT, ROCF, TMT-A/B, SDMT
Li et al. (16)	125 (*n* = 69 with cognitive follow-up)	EVT vs. IVT + EVT	ICA/M1/M2/tandem	CDR
Ettelt et al. (28)	166	IVT + EVT vs. EVT	88% anterior circulation	MoCA
Strambo et al. (25)	106	EVT vs. IVT vs. BMT	PCA (P1/P2)	Full neuropsychological battery: Praxis, visual gnosia, language, neglect, anterograde memory, executive functions, attention
López-Cancio et al. (14)	206	EVT vs. BMT	ICA/M1	TMT-A/B

ACE-R, Addenbrooke’s Cognitive Examination–Revised; ARWMC, Age-Related White Matter Changes; ASPECTS, Alberta Stroke Program Early CT Score; BA, basilar artery; BMT, best medical therapy; BNT, Boston Naming Test; BVMT, Brief Visuospatial Memory Test; CVD, cerebrovascular disease; CDR, Clinical Dementia Rating; CPM, Colored Progressive Matrices; COWAT, Controlled Oral Word Association Test; CWIT, Color–Word Interference Test; DAPT, dual antiplatelet therapy; D-KEFS, Delis–Kaplan Executive Function System; ELVO, emergent large-vessel occlusion; EVT, endovascular thrombectomy; FOIS, Functional Oral Intake Scale; FrSBe, Frontal Systems Behavior Scale; FSS, Fatigue Severity Scale; HIS, Hachinski Ischemic Score; ICA, internal carotid artery; ICA-T, terminal internal carotid artery; IVT, intravenous thrombolysis; LVO, large-vessel occlusion; MCA, middle cerebral artery; MMSE, Mini-Mental State Examination; MoCA, Montreal Cognitive Assessment; NEADL, Nottingham Extended Activities of Daily Living; NIHSS, National Institutes of Health Stroke Scale; PCA, posterior cerebral artery; RBANS, Repeatable Battery for the Assessment of Neuropsychological Status; ROCF, Rey–Osterrieth Complex Figure; SDMT, Symbol Digit Modalities Test; SNAP, Screening Neuropsychological Assessment in Stroke; TICI, Thrombolysis in Cerebral Infarction; TMT-A/B, Trail Making Test Parts A and B; TOL, Tower of London; TOPF, Test of Premorbid Functioning; WAB, Western Aphasia Battery; WAIS-IV, Wechsler Adult Intelligence Scale–IV; WMS-IV, Wechsler Memory Scale–IV.

**TABLE 2:** Cognitive outcomes after EVT and overall direction of effect.

References	Key cognitive finding	Supports EVT cognitive benefit?
Lattanzi et al. (11)	EVT combined with IVT was associated with better executive function, attention, visuospatial abilities, and memory at 6 months.	Yes
Humphrey et al. (12)	EVT was associated with better global cognition, memory, and functional outcomes, although cognitive impairment remained common.	Yes
Maglinger et al. (19)	Systemic and intracranial proteomic profiles were strongly associated with cognitive outcomes, with neurotrophic markers linked to better cognition and inflammatory markers to poorer outcomes.	Neutral (no control group)
Ospel et al. (17)	Infarct morphology was a key determinant of cognitive outcomes after EVT.	Neutral
Xu et al. (22)	Multimodal imaging-guided EVT improved neurological and cognitive outcomes at 90 days in patients with mild-to-moderate AIS.	Yes
Hazelwood et al. (29)	Inflammatory and neurotrophic biomarker profiles were associated with post-stroke cognitive outcomes.	Neutral
D’Netto et al. (20)	Cognitive and language functions improved over time, with early stroke severity predicting recovery trajectories.	Neutral
Costa Novo et al. (27)	Longer time to reperfusion was independently associated with poorer long-term cognitive outcomes.	Neutral
Li et al. (30)	Extended DAPT reduced stroke recurrence, with limited impact on cognitive outcomes.	Neutral
Bao et al. (31)	Adjunctive therapy with tirofiban and butylphthalide improved neurological recovery and reduced inflammatory markers, with modest cognitive effects.	Weak/uncertain
Ye (34)	EVT beyond the conventional time window was associated with improved cognitive outcomes and increased G-CSF expression.	Yes
Chen et al. (32)	Adjunctive tirofiban therapy was associated with reduced inflammation and improved cognitive outcomes after thrombectomy.	Yes
McLouth et al. (33)	Proteomic profiles and regional factors were associated with cognitive outcomes, with delayed EVT linked to worse recovery.	Neutral
Pu et al. (23)	EVT combined with IVT was associated with improved cognition, functional outcomes, and recanalization rates; critical-site lesions predict PSCI.	Yes
Chen et al. (24)	EVT was associated with improved global cognition and reduced inflammatory marker levels.	Yes
Regush et al. (26)	Executive and memory functions improved early after EVT, although cognitive deficits remained common.	Neutral
Joundi et al. (13)	EVT was associated with improved performance across multiple cognitive domains independent of infarct volume.	Yes
Humphrey et al. (15)	EVT was associated with better cognitive performance than best medical therapy, with similar outcomes to IVT.	Partially yes
Guglielmi et al. (21)	Cognitive impairment remained frequent despite successful recanalization and was associated with worse clinical outcomes.	Neutral
Li et al. (16)	No significant difference in cognitive outcomes was observed between direct and bridging EVT.	No
Ettelt et al. (28)	Bridging therapy was associated with improved cognitive outcomes, particularly in patients with favorable functional recovery.	Yes
Strambo et al. (25)	EVT was associated with the most favorable cognitive and visual outcomes compared with other treatment strategies.	Yes
López-Cancio et al. (14)	EVT was associated with improved executive function, particularly in patients with good functional outcomes.	Yes

AIS, acute ischemic stroke; BMT, best medical therapy; DAPT, dual antiplatelet therapy; EVT, endovascular thrombectomy; G-CSF, granulocyte colony-stimulating factor; IVT, intravenous thrombolysis; LVO, large-vessel occlusion; MMSE, Mini-Mental State Examination; MoCA, Montreal Cognitive Assessment; mRS, modified Rankin Scale; NIHSS, National Institutes of Health Stroke Scale; PSCI, post-stroke cognitive impairment.

### 3.3 Synthesis of findings

Across the 23 included studies, 11 (48%) demonstrated a cognitive advantage of EVT over intravenous thrombolysis, best medical therapy, or standard care (11–14, 22–25, 28, 32, 34). One additional study reported a partial benefit of EVT compared with conservative management but not intravenous thrombolysis (15). Ten studies (43%) yielded neutral findings, most commonly because they lacked an active comparator or focused on prognostic, imaging, or biological determinants of cognition rather than treatment effects (17, 19, 21, 26, 29–31, 33). Only one study found no cognitive difference between EVT strategies (direct versus bridging therapy) (16). Overall, a substantial proportion of the available evidence supports an association between EVT and more favorable post-stroke cognitive outcomes, although neutral findings were also common. Key cognitive findings and direction of effect are summarized in Table 2, with detailed results provided in Supplementary Table 2.

In studies directly comparing treatment strategies, EVT was generally associated with better cognitive performance. Across most comparative studies, EVT-treated patients scored on average 1–4 points higher on global cognitive screening measures such as the MoCA or MMSE at early to mid-term follow-up (12, 13, 15, 22–24, 28, 32, 34). However, the magnitude of benefit varied substantially across studies, reflecting differences in study design, baseline stroke severity, cognitive instruments, applied cut-off thresholds, and timing of assessment.

Executive function showed the most consistent domain-specific benefit, with better performance on TMT-A/B and Stroop tests more frequently observed after EVT compared with IVT or best medical therapy (11–14, 21, 25, 26). Memory outcomes were more heterogeneous, although several studies reported superior verbal and visuospatial memory performance following EVT, as assessed by RAVLT, RBANS, or WMS (11, 12, 20, 21, 26). Bridging therapy was associated with cognitive advantages in some cohorts (11, 28); however, findings were inconsistent across studies, and at least one prospective cohort reported no cognitive difference between direct and bridging EVT strategies (16). Importantly, available evidence is limited by observational designs, potential selection bias, incomplete cognitive follow-up, and heterogeneity in cognitive endpoints.

The MoCA was the most commonly used cognitive instrument (14 of 23 studies) (12, 13, 15, 19, 21, 24, 28–31, 33), with EVT generally associated with 1.5–4-point higher MoCA scores than comparator treatments (12, 13, 15, 22, 24, 28, 34). Its widespread use likely reflects sensitivity to executive, attentional, and memory deficits typical of large-vessel occlusion stroke. Nevertheless, heterogeneity in assessment timing, applied cut-off values, and the MoCA’s limited domain specificity likely contributed to the wide variability in reported rates of post-stroke cognitive impairment. A detailed overview of MoCA use across studies is provided in Supplementary Table 3.

Beyond treatment effects, several clinical and structural factors were consistently associated with poorer cognitive outcomes after EVT, including larger infarct volume, mixed gray–white matter involvement, and territorial infarct patterns (14, 17). Longer time to reperfusion independently predicted worse long-term cognitive performance (27), whereas successful recanalization was associated with better outcomes in some cohorts (23). Exploratory proteomic studies further identified inflammatory and neurotrophic markers (e.g., IGFBP3, MCP-1, ARTN) linked to cognitive trajectories after EVT (19, 29, 33). Long-term cognitive outcomes remain insufficiently characterized, with only two studies extending follow-up beyond 1 year (14, 27).

### 3.4 Risk of bias and certainty of evidence

The overall methodological quality of included studies was moderate. Most observational designs were affected by confounding, non-random treatment allocation, and incomplete adjustment for key prognostic factors (e.g., age, baseline NIHSS, infarct volume). Cognitive outcomes often relied on brief screening tools (MoCA, MMSE), and follow-up intervals varied widely, contributing to substantial heterogeneity alongside the use of diverse neuropsychological batteries.

These limitations were reflected in the formal risk-of-bias assessment. As shown in Table 3, Newcastle–Ottawa Scale scores for observational studies ranged from 3 to 7 points, with most studies scoring between 4 and 6, indicating overall moderate methodological quality. The most common sources of bias were residual confounding, retrospective or mixed data collection, and heterogeneity in cognitive assessment protocols.

**TABLE 3:** Newcastle–Ottawa Scale (NOS) risk of bias assessment.

References	Selection				Comparability		Outcome			Total quality score
	Representativeness of exposed cohort	Selection of non-exposed cohort	Ascertainment of exposure	Outcome not present at start	Adjust for most important risk factors	Adjust for other risk factors	Assessment of outcome	Follow-up length	Loss to follow-up rate	
Lattanzi et al. (11)	★	★	★	✫	★	✫	★	★	★	7/9 (moderate)
Humphrey et al. (12)	✫	✫	★	✫	✫	✫	★	★	★	4/9 (high)
Maglinger et al. (19)	★	✫	★	✫	✫	✫	★	★	★	4/9 (high)
Ospel et al. (17)	★	✫	★	✫	✫	✫	★	★	★	5/9 (moderate–high)
Xu et al. (22)	★	★	★	✫	★	✫	★	★	★	7/9 (moderate)
Hazelwood et al. (29)	★	★	★	✫	✫	✫	★	✫	★	6/9 (moderate)
D’Netto et al. (20)	✫	✫	★	✫	✫	✫	★	✫	★	3/9 (high)
Costa Novo et al. (27)	★	✫	★	✫	✫	✫	★	★	★	5/9 (moderate–high)
Li et al. (30)	★	★	★	✫	✫	✫	★	★	★	6/9 (moderate)
Bao et al. (31)	★	★	★	✫	✫	✫	★	✫	★	5/9 (moderate–high)
Chen et al. (32)	★	★	★	✫	✫	✫	★	★	★	6/9 (Moderate)
McLouth et al. (33)	★	✫	★	✫	✫	✫	★	★	★	5/9 (moderate)
Pu et al. (23)	★	★	★	✫	✫	★	★	★	★	7/9 (moderate)
Chen et al. (24)	★	★	★	✫	✫	✫	★	★	★	6/9 (moderate)
Regush et al. (26)	★	✫	★	★	✫	✫	★	★	★	6/9 (moderate)
Humphrey et al. (15)	★	✫	★	✫	✫	★	★	★	★	6/9 (moderate)
Guglielmi et al. (21)	★	✫	★	✫	✫	✫	★	✫	★	4/9 (high)
Li et al. (16)	★	★	★	✫	★	✫	★	★	✫	6/9 (moderate)
Ettelt et al. (28)	★	★	★	✫	★	✫	★	★	✫	6/9 (moderate)
Strambo et al. (25)	★	★	★	✫	★	✫	★	★	✫	6/9 (moderate)

**FIGURE 2:**
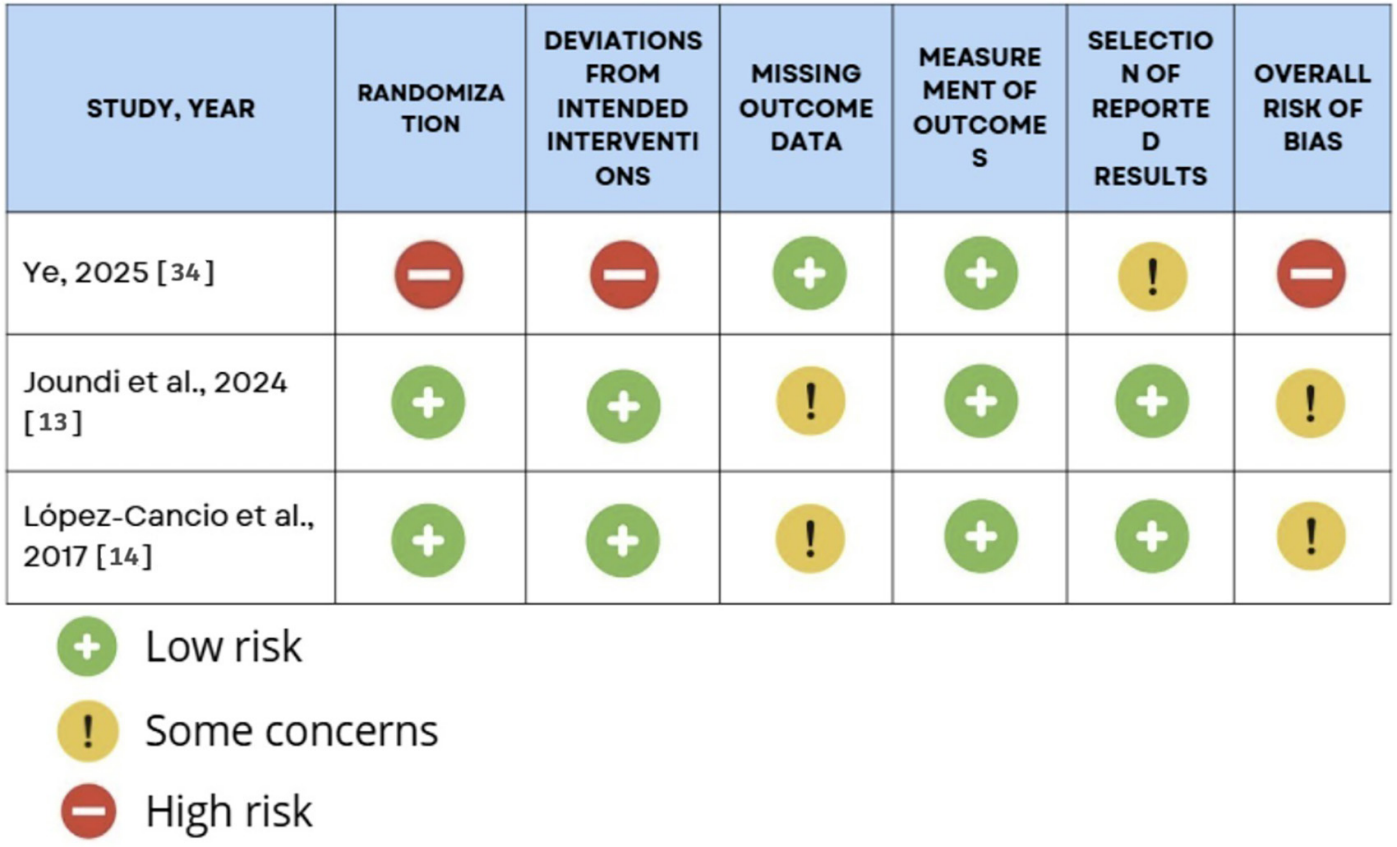
Cochrane RoB 2 risk of bias assessment for RCTs.

Randomized controlled trials also showed methodological variability. As summarized in Figure 2, RoB 2 ratings ranged from low risk to some concerns, mainly due to issues with missing cognitive outcome data and selective reporting. Most trials were not designed with cognition as a primary endpoint, resulting in limited assessor blinding, incomplete follow-up, and reliance on secondary analyses. One study was judged at high risk of bias because of deviations from intended interventions and insufficient prespecification of cognitive outcomes.

The certainty of evidence, assessed using the GRADE approach, was generally low to very low (Table 4). Improvements in global cognition and executive function were supported by low-certainty evidence, reflecting consistent benefits but downgraded for heterogeneity, indirectness, and reliance on observational designs. Evidence for memory, language, and other domain-specific outcomes was of very low certainty due to small samples and inconsistent reporting, and long-term cognitive data (>12 months) remained sparse.

**TABLE 4:** Summary of findings (GRADE): cognitive outcomes after endovascular thrombectomy.

Outcome	Effect (EVT vs. comparator)	Included studies	Participants (*n*)	Certainty (GRADE)	Comments
Global cognition (MoCA/MMSE) (3–6 months)	EVT improves MoCA/MMSE by +2 to +4 points	2 RCTs + 16 observational studies (11–13, 15–17, 19, 22–25, 27, 28, 30–34)	∼2,800	Low	Consistent benefit; downgraded for observational design and heterogeneity.
Executive function (TMT-A/B, Stroop, SDMT)	Faster TMT times, better Stroop, improved EF	1 RCT + 8 observational studies (11–14, 17, 20, 21, 25, 26)	∼2,000	Low	Strongest and most consistent domain improvement after EVT.
Memory (RAVLT, RBANS, WMS, ROCF)	Better immediate and delayed recall; improved RBANS	5 observational studies (11, 12, 20, 21, 26)	∼300	Low	Moderate, consistent effect; different tools limit comparability.
Language (AQ, WAB, BNT)	EVT associated with better language recovery	4 observational studies (12, 17, 20, 25)	∼1,200	Very low	Few studies, substantial heterogeneity.
Multidomain/neuropsychological batteries	EVT shows broad cognitive improvements across multiple domains	6 observational studies (11, 12, 20, 21, 25, 26)	∼450	Very low	Many different test batteries; not suitable for pooling.
Long-term cognition (>12 months)	Better long-term cognition associated with EVT, shorter reperfusion time, GM-only infarct	1 RCT + 1 observational study (14, 27)	250	Very low	Sparse evidence; indirectness and high heterogeneity.

AQ, Aphasia Quotient; BNT, Boston Naming Test; EF, executive function; EVT, endovascular thrombectomy; GM, gray matter; MMSE, Mini-Mental State Examination; MoCA, Montreal Cognitive Assessment; RCT, randomized controlled trial; RBANS, Repeatable Battery for the Assessment of Neuropsychological Status; RAVLT, Rey Auditory Verbal Learning Test; ROCF, Rey–Osterrieth Complex Figure; SDMT, Symbol Digit Modalities Test; TMT-A/B, Trail Making Test Parts A and B; WAB, Western Aphasia Battery; WMS, Wechsler Memory Scale.

## 4 Discussion

This systematic review synthesized evidence from 23 studies evaluating cognitive outcomes after EVT for acute ischemic stroke. Although EVT is well established for improving survival and functional independence (3, 4), its cognitive effects have remained less clearly defined. Overall, available evidence indicates that EVT is generally associated with more favorable cognitive outcomes than IVT, best medical therapy, or standard care, with MoCA/MMSE scores typically 1–4 points higher in EVT-treated patients (11–13, 15, 22, 24, 34). Nonetheless, post-stroke cognitive impairment remained common, consistent with the high prevalence of cognitive deficits after ischemic stroke (5, 7). This review provides an integrated synthesis of cognitive, neuropsychological, imaging, and proteomic findings relevant to cognition after EVT.

Across studies, EVT consistently yielded better global cognitive performance at early and mid-term follow-up. Although the observed differences in MoCA/MMSE scores were relatively small (typically 1–4 points), they may still be clinically meaningful and translate into improvements in independence, participation, and quality of life (6). Even small changes in global cognitive screening scores can reflect differences in executive functioning, attention, and processing speed that are not fully captured by functional scales such as the modified Rankin Scale (7). Importantly, a change of approximately 1.22–2.15 points in MoCA has been estimated as the minimal clinically important difference in stroke rehabilitation populations (35). Moreover, cognitive deficits–particularly in executive function–are strongly associated with reduced independence in instrumental activities of daily living, return to work, and overall quality of life after stroke (7, 9).

Some studies reported additional cognitive benefits of bridging therapy compared with IVT alone or direct EVT (11, 28), potentially reflecting enhanced microvascular reperfusion with IVT (2), whereas others found no difference between the two EVT approaches (16), highlighting the need for trials specifically powered for cognitive endpoints. However, current evidence comparing bridging and direct EVT with respect to cognitive outcomes remains inconclusive. Observational registry data suggesting a cognitive advantage of bridging therapy (28) contrast with prospective cohort findings showing no significant difference between strategies (16), and both are limited by residual confounding, selection bias, heterogeneous cognitive endpoints, and incomplete long-term follow-up.

Executive function showed the most consistent improvement, with faster TMT-A/B performance and better Stroop or SDMT scores after EVT (11–14, 26), supporting the notion that timely reperfusion helps preserve fronto-subcortical networks vulnerable to ischemia. Memory outcomes were more heterogeneous, though several studies reported gains in verbal learning, delayed recall, and working memory (11, 12, 20, 21, 26). Language recovery, less frequently evaluated, also improved in some EVT-treated cohorts (12, 20, 25). Despite these benefits, PSCI remained frequent–affecting 60%–77% of individuals after EVT (12, 21)–indicating that reperfusion alone does not fully prevent cognitive decline, which also depends on white-matter integrity, network connectivity, and neurobiological recovery. These domain-specific patterns raise the question of the underlying mechanisms through which EVT may influence cognitive recovery.

Beyond these descriptive findings, several mechanisms may help explain the observed cognitive benefits associated with EVT. Rapid and successful reperfusion likely limits infarct core expansion and preserves structurally strategic cortical and subcortical regions involved in higher-order cognitive functions, including attention, executive function, memory, and language (14, 17, 27). This may be particularly relevant in large-vessel occlusion, where untreated ischemia can disrupt distributed fronto-subcortical and cortico-cortical networks essential for cognition. In addition, EVT may reduce secondary injury by limiting white-matter tract damage and network disconnection, which are increasingly recognized as key contributors to post-stroke cognitive impairment (7, 17). This network-preserving effect may partly explain why executive functions–highly dependent on distributed connectivity–showed the most consistent improvement across studies. Furthermore, faster reperfusion may attenuate inflammatory cascades and delayed neuronal injury, both of which have been linked to worse cognitive outcomes after stroke. The proteomic findings identified in several included studies support this interpretation, as pro-inflammatory markers (e.g., MCP-1) were associated with poorer cognition, whereas neurotrophic or vascular-repair pathways were linked to more favorable recovery (19, 29, 33). Taken together, these observations suggest that the cognitive benefit of EVT is not solely a consequence of improved global functional outcome, but may also reflect preservation of brain networks specifically relevant to cognition.

Structural, clinical, and biological determinants of cognition were consistently identified. Mixed gray–white matter involvement, larger infarct size, and territorial lesion patterns were associated with worse cognitive outcomes (14, 17), reinforcing network-based models of PSCI (7). Shorter time to reperfusion predicted better cognition (27), underscoring that “time is brain” applies to cognitive as well as functional outcomes (2, 4). Proteomic studies further highlighted inflammatory and neurotrophic markers (e.g., IGFBP3, MCP-1, ARTN, HGF, TIE1) linked to cognitive trajectories (19, 29, 33), with pro-inflammatory profiles associated with poorer outcomes and trophic or vascular-repair pathways linked to recovery, suggesting a role for biomarker-informed stratification.

From a clinical perspective, these findings suggest that cognitive outcomes should be considered alongside traditional functional endpoints after EVT. Even among patients with favorable motor recovery, cognitive deficits may remain substantial and clinically relevant. Routine post-stroke cognitive screening–preferably using tools sensitive to executive dysfunction and, when feasible, followed by domain-specific neuropsychological assessment–may improve identification of patients requiring further rehabilitation or follow-up. Future research should prioritize standardized cognitive outcome sets, prespecified cognitive endpoints in EVT trials, and longer-term follow-up beyond 12 months. Comparative studies of direct versus bridging EVT, as well as studies integrating imaging markers of structural connectivity and biomarker-based profiling, may further clarify which patients derive the greatest cognitive benefit from reperfusion therapy.

This review has several limitations: most included studies were observational, with risks of selection bias, confounding, and heterogeneous cognitive assessments. Existing RCTs did not prespecify cognition as a primary endpoint, resulting in incomplete follow-up and limited blinding (13, 14, 34). Long-term cognitive outcomes were rarely reported (14, 27), and variability in neuropsychological batteries, imaging measures, and biomarker protocols limited comparability across studies. In addition, only English-language studies were included, which may have introduced language bias and led to the exclusion of relevant studies published in other languages. Furthermore, due to substantial heterogeneity in cognitive outcome definitions, assessment tools, follow-up intervals, and reporting of effect estimates, a quantitative meta-analysis was not feasible. As a result, the findings are based on narrative synthesis, which limits the precision of the overall estimates of cognitive benefit. Despite these constraints, no study indicated cognitive harm from EVT, and many demonstrated clear benefit over non-EVT strategies.

## 5 Conclusion

In this systematic review, EVT was associated with more favorable cognitive outcomes than IVT, best medical therapy, or standard care, particularly in global cognition and executive function, although persistent post-stroke cognitive impairment remained common. Better outcomes were most consistently linked to timely reperfusion, successful recanalization, and less extensive infarct patterns. However, the overall certainty of evidence remains low due to observational study designs, heterogeneous cognitive assessments, and limited long-term follow-up. Future studies should incorporate standardized cognitive endpoints and longer follow-up to better define the role of EVT in cognitive recovery.

## Data Availability

The original contributions presented in this study are included in the article/Supplementary material, further inquiries can be directed to the corresponding author.
