# Supplementary material for: Cognitive outcomes after endovascular thrombectomy in ischemic stroke: a systematic review
Source: Front Med (Lausanne). 2026 May 11;13:1787129. doi: 10.3389/fmed.2026.1787129 (PMC13199237; doi:10.3389/fmed.2026.1787129)
Supplement: Supplementary file 1 [file Table_1.docx]

Supplementary Material 1

A comprehensive search of four electronic databases (PubMed, Embase, Scopus, and the Cochrane Library) was performed between 1 March 2025 and 31 October 2025.

The strategy was developed according to the Preferred Reporting Items for Systematic Reviews and Meta-Analyses (PRISMA 2020) and PRISMA-S guidelines.

The search combined three main concepts:

1. Ischemic stroke
2. Mechanical thrombectomy
3. Cognitive outcomes or neuropsychological assessment

Both controlled vocabulary (MeSH, Emtree) and free-text terms were used.

The search was limited to studies published in English, involving human adults, between 1 January 2015 and 31 October 2025.

All results were exported to Rayyan for deduplication and screening.

Notes:

- All search strings were copied verbatim from each database’s advanced-search interface.
- No restrictions were applied regarding study design.
- Reference lists of included articles were screened manually to identify additional relevant studies.
- Search strategies were reviewed for completeness and logical consistency in accordance with the PRESS (Peer Review of Electronic Search Strategies) guideline.

**Supplementary Table S1.** Search strategies and results

| **Database** | **Search string (verbatim)** | **Filters/limits** | **Records retrieved** |
| --- | --- | --- | --- |
| **PubMed** | ( "Stroke, Ischemic"[MeSH Terms] OR "Brain Ischemia"[MeSH Terms] OR "ischemic stroke"[tiab] OR "cerebral infarction"[tiab] OR "brain ischemia"[tiab] ) AND ( "Thrombectomy"[MeSH Terms] OR "mechanical thrombectomy"[tiab] OR "endovascular thrombectomy"[tiab] OR "endovascular treatment"[tiab] OR "endovascular therapy"[tiab] ) AND ( ("Cognition"[MeSH Terms] OR "Cognition Disorders"[MeSH Terms] OR "Neuropsychological Tests"[MeSH Terms] OR cognition[tiab] OR cognitive[tiab] OR neurocognit*[tiab] OR "cognitive impairment"[tiab] OR "neuropsychological outcome*"[tiab] OR "neuropsychological assessment"[tiab]) OR ("Mini-Mental State Examination"[tiab] OR MMSE[tiab] OR "Montreal Cognitive Assessment"[tiab] OR MoCA[tiab] OR "Trail Making Test"[tiab] OR TMT[tiab] OR Stroop[tiab] OR "Digit Span"[tiab] OR "Symbol Digit Modalities Test"[tiab] OR SDMT[tiab] OR "Rey Auditory Verbal Learning Test"[tiab] OR RAVLT[tiab] OR "California Verbal Learning Test"[tiab] OR CVLT[tiab] OR "Hopkins Verbal Learning Test"[tiab] OR HVLT[tiab] OR "Rey-Osterrieth Complex Figure"[tiab] OR "Wisconsin Card Sorting Test"[tiab] OR WCST[tiab] OR "Controlled Oral Word Association Test"[tiab] OR COWAT[tiab] OR "Verbal Fluency"[tiab] OR "semantic fluency"[tiab] OR "category fluency"[tiab] OR FAS[tiab] OR "Boston Naming Test"[tiab] OR "Addenbrooke's Cognitive Examination"[tiab] OR ACE[tiab] OR CERAD[tiab] OR CANTAB[tiab] OR "Clock Drawing Test"[tiab] OR "Frontal Assessment Battery"[tiab] OR FAB[tiab]) ) | English; Humans; 2015–2025 | **129** |
| **Embase** | ('ischemic stroke'/exp OR 'ischemic stroke':ti,ab OR 'cerebral infarction':ti,ab OR 'brain ischemia':ti,ab) AND ('thrombectomy'/exp OR 'mechanical thrombectomy':ti,ab OR 'endovascular thrombectomy':ti,ab OR 'endovascular treatment':ti,ab OR 'endovascular therapy':ti,ab) AND (('cognition'/exp OR 'cognitive function':ti,ab OR 'neurocognitive':ti,ab OR 'cognitive impairment':ti,ab OR 'memory':ti,ab OR 'executive function':ti,ab OR 'neuropsychological':ti,ab) OR ('mini mental state examination':ti,ab OR MMSE:ti,ab OR 'montreal cognitive assessment':ti,ab OR MoCA:ti,ab OR 'trail making test':ti,ab OR TMT:ti,ab OR 'stroop test':ti,ab OR Stroop:ti,ab OR 'digit span':ti,ab OR 'symbol digit modalities test':ti,ab OR SDMT:ti,ab OR 'rey auditory verbal learning test':ti,ab OR RAVLT:ti,ab OR 'california verbal learning test':ti,ab OR CVLT:ti,ab OR 'hopkins verbal learning test':ti,ab OR HVLT:ti,ab OR 'wisconsin card sorting test':ti,ab OR WCST:ti,ab OR 'controlled oral word association test':ti,ab OR COWAT:ti,ab OR 'verbal fluency':ti,ab OR 'semantic fluency':ti,ab OR 'category fluency':ti,ab OR 'boston naming test':ti,ab OR 'addenbrooke* cognitive examination':ti,ab OR ACE:ti,ab OR CERAD:ti,ab OR CANTAB:ti,ab OR 'clock drawing test':ti,ab OR 'frontal assessment battery':ti,ab OR FAB:ti,ab)) | English; 2015–2025 | **1,575** |
| **Scopus** | TITLE-ABS-KEY ( "ischemic stroke" OR "cerebral infarction" OR "brain ischemia" ) AND TITLE-ABS-KEY ( "thrombectomy" OR "mechanical thrombectomy" OR "endovascular thrombectomy" OR "endovascular treatment" OR "endovascular therapy" ) AND TITLE-ABS-KEY ( "cognition" OR "cognitive function" OR "neurocognitive" OR "cognitive impairment" OR "memory" OR "executive function" OR "neuropsychological" ) OR TITLE-ABS-KEY ( "Mini Mental State Examination" OR MMSE OR "Montreal Cognitive Assessment" OR MoCA OR "Trail Making Test" OR TMT OR Stroop OR "Digit Span" OR "Symbol Digit Modalities Test" OR SDMT OR "Rey Auditory Verbal Learning Test" OR RAVLT OR "Hopkins Verbal Learning Test" OR HVLT OR "Wisconsin Card Sorting Test" OR WCST OR "Controlled Oral Word Association Test" OR COWAT OR "Verbal Fluency" OR "semantic fluency" OR "category fluency" OR "Boston Naming Test" OR "Addenbrooke* Cognitive Examination" OR ACE OR CERAD OR CANTAB OR "Clock Drawing Test" OR "Frontal Assessment Battery" OR FAB OR "California Verbal Learning Test" OR CVLT ) | English; 2015–2025 | **432** |
| **Cochrane Library** | (ischemic stroke OR cerebral infarction OR brain ischemia) AND (thrombectomy OR mechanical thrombectomy OR endovascular thrombectomy OR endovascular treatment OR endovascular therapy) AND (cognition OR cognitive OR neurocognitive OR cognitive impairment OR neuropsychological OR mini mental OR MMSE OR montreal cognitive OR MoCA OR trail making OR TMT OR stroop OR digit span OR symbol digit OR SDMT OR rey auditory OR RAVLT OR hopkins verbal OR HVLT OR wisconsin card OR WCST OR verbal fluency OR semantic fluency OR category fluency OR boston naming OR addenbrooke OR ACE OR CERAD OR CANTAB OR clock drawing OR frontal assessment OR FAB OR california verbal OR CVLT) | English; 2015–2025 | **87** |
