# Supplementary material for: Cognitive outcomes after endovascular thrombectomy in ischemic stroke: a systematic review
Source: Front Med (Lausanne). 2026 May 11;13:1787129. doi: 10.3389/fmed.2026.1787129 (PMC13199237; doi:10.3389/fmed.2026.1787129)
Supplement: Supplementary file 2 [file Table_2.docx]

Supplementary Material 2

**Table S2.** Cognitive Outcomes After EVT and Overall Direction of Effect **Abbreviations:** ADL – activities of daily living; AIS – acute ischemic stroke; ANG – angiogenin; APOM – apolipoprotein M; ARTN – artemin; ASPECTS – Alberta Stroke Program Early CT Score; AQ – Aphasia Quotient; BI – Barthel Index; BMT – best medical therapy; CDR – Clinical Dementia Rating; CI – confidence interval; COMP – cartilage oligomeric matrix protein; DAPT – dual antiplatelet therapy; DNER – Delta/notch-like EGF-related receptor; ELVO – emergent large-vessel occlusion; EVT – endovascular thrombectomy; G-CSF – granulocyte colony-stimulating factor; GM/WM – gray/white matter; GP1BA – glycoprotein Ib alpha chain; HGF – hepatocyte growth factor; hs-CRP – high-sensitivity C-reactive protein; ICA – internal carotid artery; IGFBP3 – insulin-like growth factor binding protein 3; IVT – intravenous thrombolysis; LIFR – leukemia inhibitory factor receptor; LVO – large-vessel occlusion; MBI – Modified Barthel Index; MBP – myelin basic protein; MCP-1 – monocyte chemoattractant protein-1; MMSE – Mini-Mental State Examination; MoCA – Montreal Cognitive Assessment; mRS – modified Rankin Scale; NIHSS – National Institutes of Health Stroke Scale; NSE – neuron-specific enolase; PSCI – post-stroke cognitive impairment; PTPRS – protein tyrosine phosphatase receptor S; RAVLT – Rey Auditory Verbal Learning Test; RBANS – Repeatable Battery for the Assessment of Neuropsychological Status; ROCF – Rey–Osterrieth Complex Figure; SDMT – Symbol Digit Modalities Test; SMC – standard medical care; SNAP – Screening Neuropsychological Assessment in Stroke; TICI – Thrombolysis in Cerebral Infarction; TIE1 – tyrosine kinase with immunoglobulin-like and EGF-like domains 1; TMT-A/B – Trail Making Test A/B.

| **Study (Year)** | **Main Cognitive Outcomes (Effect Size/ Statistics)** |
| --- | --- |
| Lattanzi et al., 2020 [11] | Stroop WR adjβ=13.99 (p<0.001); TMT-A adjβ=−92.98 (p=0.003); RAVLT-I adjβ=12.60 (p<0.001). |
| Humphrey et al., 2024 (JINS) [12] | MoCA adjβ=2.14 (p=0.002); RAVLT Total β=8.26 (p=0.007); Symbol Span β=1.62 (p=0.009); d=0.507. |
| Maglinger et al., 2023 [19] | Discharge MoCA: positive correlations with systemic & intracranial proteins (e.g., DNER R²=0.42, p=0.001; APOM R²=0.33, p=0.004; IGFBP3 R²=0.29, p=0.008).  90-day MoCA: negative correlations: ARTN R²=0.59, p=0.002; MCP-1 R²=0.56, p=0.003; COMP R²=0.46, p=0.007.  Positive correlations: IGFBP3 R²=0.45, p=0.01; TIE1 R²=0.43, p=0.02. |
| Ospel, et al., 2024 [17] | MoCA worse with ↑infarct volume (aCOR=1.05/10mL), GM+WM (aCOR=1.92), WM volume (aCOR=1.36), territorial pattern (aCOR=1.65). |
| Xu et al., 2017 [22] | MoCA +1.61 (p=0.022); MMSE +1.55 (p=0.023); BI>95: 65.6% vs 28.6% (p=0.005); mRS 0–2: 62.5% vs 33.3% (p=0.024). |
| Hazelwood et al., 2022 [29] | MoCA correlations: MoCA negatively correlated with ARTN (r=−0.772), HGF (r=−0.642). MoCA positively correlated with GP1BA (r=+0.600), CCL5/CXCL5 cluster. |
| D'Netto, et al., 2024 [20] | Language: AQ improved significantly T1→T2 (p=0.002), T2→T3 (p=0.021). At T1 71% aphasia; at T3 persistent aphasia in 45%.  Cognition: RBANS total improved T1→T2 (p=0.001), T1→T3 (<0.001). Cognitive impairment: T1 80%, T2 52%, T3 39%.  Executive function: TMT-A/B improved significantly; Brixton NS.  24h NIHSS strongly correlated cognition at T1, T2, T3 (e.g., AQ T1 r = –0.748, p < 0.05). |
| Costa Novo et al., 2024 [27] | Reperfusion time: β=−0.0207 (p=0.020); education strongly protective (p<0.0001). |
| Li et al., 2024 [30] | MoCA improvement NS (p=0.080). AIS recurrence lower in 3-mo DAPT (p=0.047). |
| Bao et al., 2025 [31] | MMSE/MoCA ↑ in treatment arm but NS; inflammatory markers significantly lower (all p<0.05). |
| Ye, 2025 [34] | MoCA higher in EVT at 72 h and 90 d (both p=0.001). G-CSF correlated with MoCA (r=0.559). |
| Chen et al. 2025 [32] | MMSE (2 weeks): significantly higher in MT+T (p < 0.001)  • MBI, FMA: greater improvement in MT+T (p < 0.001)  • NSE, S100β, MBP: lower after MT+T (p < 0.001)  • hs-CRP, TNF-α, IL-6: more reduced in MT+T (p < 0.001) |
| McLouth et al., 2024 [33] | Significant protein–outcome interactions: NIHSS (CXCL9 p=0.021, THBS4 p=0.022, CXCL10 p=0.027, IL15RA p=0.027, IL13 p=0.028, PRSS2 p=0.028, IL6 p=0.031); MoCA (ANG p=0.037, LIFR p=0.046); mRS (IL13 p=0.003; PTPRS p=0.031; CCL25 p=0.039) |
| Pu et al., 2023 [23] | MMSE higher in EVT+IVT (p<0.001); mRS improved (p<0.01); recanalization 90.6% vs 76%. |
| Chen et al., 2021 [24] | MMSE D90: 27.68±1.87 vs 23.54±1.79 (p<0.001); MoCA D90: 26.87±2.28 vs 22.06±1.62 (p<0.001). |
| Regush et al., 2025 [26] | TMT-A improved (p<0.001); Stroop I & II improved (p<0.001); Luria improved (p<0.05). |
| Joundi et al., 2024 [13] | MoCA aOR 2.32; SNAP aOR 3.85; Trails A aOR 3.50; Trails B aOR 2.56 + global binary aOR 2.57; global ordinal aOR 2.83 (5-test composite). |
| Humphrey et al., 2024 (Brain Impair.) [15] | MoCA: EVT 24.1; IVT 23.9; BMT 20.7 (ANOVA p=0.012). |
| Guglielmi et al., 2023 [21] | MoCA: 10.19 (CImp) vs 22.35 (noCImp) (p<0.001). Lesion extent correlates with worse attention/executive function. |
| Li et al., 2023 [16] | Poststroke dementia (CDR ≥1): Direct 42.1% vs Bridging 22.6%, p = 0.12 (NS). Ordinal regression: no association between treatment and CDR outcome (OR 1.65, p=0.26) |
| Ettelt et al., 2020 [28] | Bridging +2.39 MoCA (p=0.033). |
| Strambo et al., 2020 [25] | EVT vs. BMT:  • Recanalization: OR 4.11 (1.35–12.53)  • Cognitive outcome: OR 5.22 (1.37–19.9)  • Visual field normalization: OR 2.94 (0.96–9.05).  IVT vs. BMT:  • Recanalization: OR 10.62 (2.13–52.92)  • Cognitive outcome: OR 2.21 (0.52–9.34) (NS) |
| López-Cancio et al., 2017 [14] | TMT-B completion in due time:  • 3 mo: aOR 3.17 (1.51–6.66)  • 1 yr: aOR 3.66 (1.60–8.35)  TMT-A fewer errors: aOR 2.45 (1.05–5.70)  TMT-A/B faster times (p=0.047–0.030 among mRS≤2) |
