# Supplementary material for: Cognitive outcomes after endovascular thrombectomy in ischemic stroke: a systematic review
Source: Front Med (Lausanne). 2026 May 11;13:1787129. doi: 10.3389/fmed.2026.1787129 (PMC13199237; doi:10.3389/fmed.2026.1787129)
Supplement: Supplementary file 3 [file Table_3.docx]

Supplementary Material 3

**Supplementary Table S3.** Studies Using the Montreal Cognitive Assessment (MoCA) for Cognitive Outcomes After Endovascular Thrombectomy
Abbreviations: EVT —endovascular thrombectomy; IVT —intravenous thrombolysis; BMT —best medical therapy; NR —not reported.

| **Study (Reference)** | **Participants assessed with MoCA (n)** | **Intervention / Comparator** | **Cognitive Timing** | **Key MoCA Findings** |
| --- | --- | --- | --- | --- |
| Humphrey et al., 2024 (JINS) [12] | 62 | EVT vs Standard Medical Care (IVT + conservative) | 90 days | EVT: higher MoCA (adj β=2.14, p=0.002) |
| Maglinger et al., 2023 [19] | 28 | EVT | 90 days | MoCA correlated with proteomic markers (DNER+, APOM+, IGFBP3+) |
| Ospel et al., 2024 [17] | 706 | EVT | 90 days | Worse MoCA with larger infarct volume, WM involvement |
| Xu et al., 2017 [22] | 90 | EVT vs. IVT | 90 days | EVT: higher MoCA (26.23 vs 24.62, p=0.022) |
| Hazelwood et al., 2022 [29] | 61 | EVT vs. CVD controls | 90 days | MoCA correlated negatively with ARTN, HGF; positively with GP1BA |
| Li et al., 2024 [30] | 147 | 1 months vs. 3 months DAPT after MT | 90 days | MoCA: NS improvement, p=0.080 |
| Bao et al., 2025 [31] | 73 | EVT + tirofiban + butylphthalide vs EVT | 90 days | Small NS improvement in MoCA |
| Ye, 2025 [34] | 60 | EVT vs IVT (>6h) | 72 h, 90 days | EVT MoCA higher at 72h & 90d (p=0.001) |
| McLouth et al., 2024 [33] | 81 | EVT | 90 days | MoCA associated with ANG and LIFR |
| Chen et al., 2021 [24] | 169 | EVT+SC vs SC | 90 days | EVT MoCA: 26.87 vs 22.06 (p<0.001) |
| Joundi et al., 2024 [13] | 315 | EVT vs BMT | 90 days | EVT improved MoCA (favourable outcome aOR 2.32) |
| Humphrey et al., 2024 (Brain Impair.) [15] | 82 | EVT vs IVT vs BMT | NR | EVT MoCA highest (p=0.012) |
| Guglielmi et al., 2023 [21] | 43 | EVT | NR | Lower MoCA in cognitively impaired subgroup |
| Ettelt et al., 2020 [28] | 166 | Bridging IVT + EVT vs EVT | 90 days | Bridging +2.39 MoCA (p=0.033) |
